# Association between Social Isolation and Loneliness with Estimated Atherosclerotic Cardiovascular Disease Risk in a UK Biobank Population-Based Study

**DOI:** 10.3390/ijerph20042869

**Published:** 2023-02-06

**Authors:** Alexandre Vallée

**Affiliations:** Department of Epidemiology-Data-Biostatistics, Delegation of Clinical Research and Innovation (DRCI), Foch Hospital, 92150 Suresnes, France; al.vallee@hopital-foch.com

**Keywords:** social isolation, loneliness, gender, education, income, cardiovascular disease, cardiovascular risk, atherosclerosis, atherosclerotic cardiovascular disease

## Abstract

Objective: The association of cardiovascular (CV) risk with social isolation and loneliness remains poorly studied. The purpose of this cross-sectional study was to investigate the associations between social isolation and loneliness with estimated 10-year atherosclerotic cardiovascular disease (ASCVD) risk. Methods: Among 302,553 volunteers of the UK Biobank population, social isolation and loneliness were assessed with a questionnaire. Associations between social isolation and loneliness with ASCVD risk were estimated using multiple gender regressions. Results: Men presented a higher estimated 10-year ASCVD risk (8.63% vs. 2.65%, *p* < 0.001) and higher proportions of social isolation (9.13% vs. 8.45%, *p* < 0.001) and loneliness (6.16% vs. 5.57%, *p* < 0.001) than women. In all covariate-adjusted models, social isolation was associated with an increased ASCVD risk in men (*B* = 0.21 (0.16; 0.26), *p* < 0.001) and women (*B* = 0.12 (0.10; 0.14), *p* < 0.001). Loneliness was associated with an increased ASCVD risk in men (*B* = 0.08 (0.03; 0.14), *p* = 0.001) but not in women (*p* = 0.217). A significant interaction was observed between social isolation and loneliness with ASCVD risk in men (*p* = 0.009) and women (*p* = 0.016). After adjustment for all covariates, both social isolation and loneliness were significantly associated with ASCVD risk in men (*B* = 0.44 (0.28; 0.61), *p* < 0.001) and women (*B* = 0.20 (0.12; 0.29), *p* < 0.001). Conclusion: Social isolation was associated with a higher estimated 10-year ASCVD risk in both genders but only loneliness among men. Social isolation and loneliness can be considered potential added risk factors for CV risk. Health policies should address these notions in prevention campaigns, in addition to traditional risk factors.

## 1. Introduction

Cardiovascular (CV) disease is one of the main causes of morbidity and mortality worldwide [1]. The impact of CV disease is major in terms of economic burden [2]. Understanding and identifying non-traditional risk factors could improve preventive health strategies. In 2016, a systematic review showed that participants with poor social determinants had a 30% more likely risk of cardiac and stroke events [3]. Even if no established definition exists, social isolation and loneliness are associated with the ability of individuals to form satisfying and meaningful relationships and social interactions and to be adaptative in social situations and interactions with other people, services and institutions [4]. Social isolation is a measure of the absence of social interactions or infrequent social contact with other people. Loneliness is the subjective negative notion of feeling isolated [5]. In recent years, social isolation has become a major public health issue [6]. The association between CV disease and social isolation and loneliness remains inconsistent, with studies showing no association [7], association with non-fatal CV events [8], or associations with CV events [4,7,9,10]. However, few studies have focused on large populations [11]. The link between social isolation and CV disease could be modulated by the atherosclerotic pathway [12]. To date, it remains unclear if these relationships are independent of biological factors [13]. Thus, in this cross-sectional study, I evaluated estimated 10-year atherosclerotic cardiovascular disease (ASCVD) risk [14,15] to investigate social isolation and loneliness associations among the general UK Biobank population.

## 2. Methods

### 2.1. UK Biobank Population

The UK Biobank cohort comprised 9.1 million eligible individuals, 8.6 million of which did not respond or did not provide consent. Thus, at baseline, the UK Biobank included 502,478 Britons (5.5% of the total UK Biobank cohort), aged 38–73 years, across 22 UK cities from the UK National Health Service Register between 2006 and 2010, 90 M of which were linked to national health registries. Participants responded to questionnaires and a computer-assisted interview, and they were subject to physical and functional measures and blood, urine, and saliva sampling [16]. Data included personalized information of the participants, including socio-economic, behavior and lifestyle, mental health battery, clinical diagnoses and therapies, genetics, imaging and physiological biomarkers from blood and urine samples. The cohort protocol can be found in the literature [17,18].

### 2.2. Ethical Considerations

All participants provided electronic informed consent, and the UK Biobank received ethical approval from the Northwest Multi-center Research Ethics Committee (MREC) covering the whole of the UK. The study was conducted according to the guidelines of the Declaration of Helsinki and approved by the Northwest Haydock Research Ethics Committee (protocol code: 21/NW/0157, date of approval: 21 June 2021). For details, see https://www.ukbiobank.ac.uk/learn-more-about-uk-biobank/about-us/ethics (Accessed on 30 November 2022).

### 2.3. Study Population

**Figure 1:**
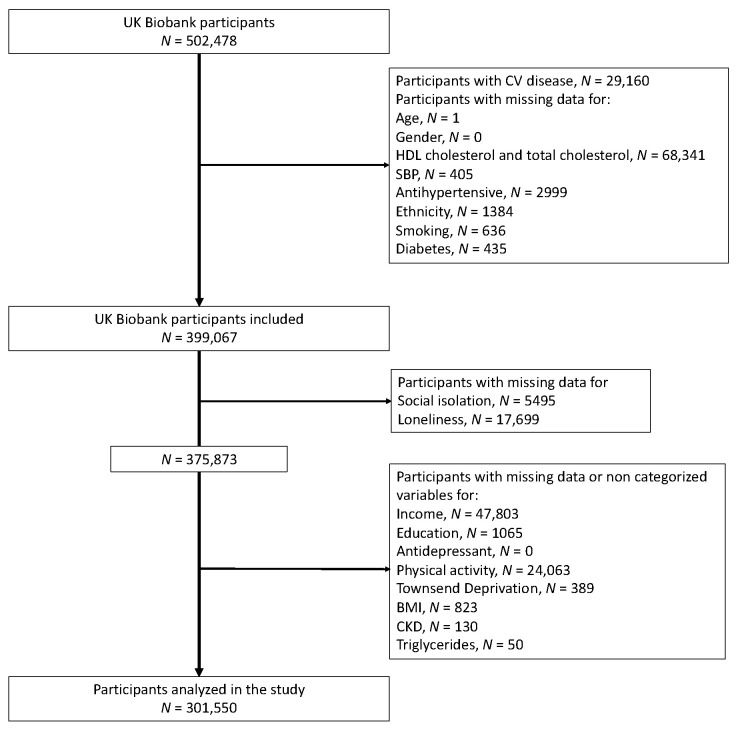
Flowchart.

We included 399,067 volunteers of the UK Biobank without missing data and without previous CV events to calculate the estimated 10-year ASCVD risk. CV diseases were defined as including heart attack, angina, and stroke, as diagnosed by a doctor and reported in questionnaires. Of these, I excluded 97,517 for missing data. We therefore analyzed the data of 301,550 volunteers (Figure 1).

### 2.4. Estimated 10-Year ASCVD Risk

Estimated 10-year ASCVD risk was evaluated using the Pooled Cohort Equations (PCE) model [14,15]. The PCE model was used to express ASCVD risk in a continuous percentage. The estimated 10-year absolute risk of ASCVD was set to be characterized by death due to coronary heart disease, nonfatal myocardial infarction or fatal or nonfatal stroke over a 10-year period in participants free of established CV diseases. The PCE allowed for the derivation of sex- and race-specific estimates of the 10-year risk for ASCVD for adults aged 40 to 79 years. Parameters included in the PCE were age, gender, Black people, tobacco smoking, total and high-density lipoprotein cholesterol, treated or untreated systolic blood pressure, and diabetes. A PCE score of 7.5% or greater indicated that a participant was at a high ASCVD risk, and participants with a PCE score of less than 7.5% were considered at low risk [19,20].

### 2.5. Social Isolation and Loneliness

Social isolation and loneliness were assessed with scales that were used in previous UK Biobank studies [7,9,11].

The social isolation scale contained three questions (1) “Including yourself, how many people are living together in your household?”; (2) “How often do you visit friends or family or have them visit you?”; and (3) “Which of the following (leisure/social activities) do you engage in once a week or more often? You may select more than one”), where certain answers were given one point (1 point for no participation in social activities at least weekly, 1 point for living alone, and 1 point for friend and family visits less of than once a month), and all other answers were given 0 point. This resulted in a scale ranging from 0 to 3, where a person was defined as socially isolated if she/he had two or more points.

Loneliness was measured with two questions: “Do you often feel lonely?” (no = 0; yes = 1) and “How often are you able to confide in someone close to you?” (0 = almost daily to once every few months; 1 = never or almost never). An individual was defined as lonely if she/he answered positively to both questions (score 2).

### 2.6. Covariates

Systolic and diastolic blood pressure (SBD, DBP) were measured twice at the assessment center with an automated BP device (Omron 705 IT electronic blood pressure monitor; OMRON Healthcare Europe B.V. Kruisweg 577 2132 NA Hoofddorp) or manually with a sphygmomanometer equipped with an inflatable cuff in association with a stethoscope if the blood pressure device failed to measure the BP or if the largest inflatable cuff of the device did not fit around the individual’s arm [21].

Diabetes status was defined on either receiving anti-diabetic medication, diabetes diagnosed by a doctor, or a fasting glucose concentration of ≥7 mmol/L [22]. Medications were characterized by the question: “Do you regularly take any of the following medications?”.

The biological parameters are detailed in the UK Biobank protocol [23].

The estimated glomerular filtration rate (eGFR) was calculated based on the Chronic Kidney Disease Epidemiology Collaboration equation (eGFR-CKD-EPI), as follows:

eGFR=141×(minimum of 1 or standardizedScrκ)α ×(maximum of 1 or standardizedScrκ)−1.209 ×[0.993]^age×(1.018 if female)

where κ is 0.7 in females and 0.9 in males and α is −0.329 in females and −0.411 in males. eGFR <60 mL/min/1.73 m^2^ indicated chronic kidney disease (CKD)).

The body mass index was calculated as weight (in kg) divided by height^2^ (meters).

Education level was defined in three categories: high (college or university degree), intermediate (A/AS levels or equivalent, O levels/GCSEs or equivalent, or other professional qualifications such as nursing and teaching), and low (none of the afore mentioned categories).

Income level was defined as: high level (greater than *£*52,000 per year), moderate level (between *£*18,000 and *£*51,999 per year), and low level (less than *£*18,000 per year).

Townsend deprivation index scores were derived from national census data about car ownership, household overcrowding, owner occupation, and unemployment aggregated for postcodes of residence [24].

Current tobacco smokers were defined as participants who responded “yes, on most or all days” or “yes, only occasionally” at the question “do you smoke tobacco now”.

Antidepressant medication use was included in the analyses due to the association between depression and social isolation [25]. The list of antidepressant drugs is available at [26].

Physical activity was self-reported, measured with a revised version of the International physical activity questionnaire (IPAQ) [27] completed on a tablet computer during examination. Patients were asked to state how many days they were engaged in more than 10 min of walking, moderate physical activity, and vigorous physical activity in a typical week. Individuals were then asked for how many minutes they were engaged in each of the activities on a typical day. Self-reported physical activity data were processed using the method of Bradbury et al. [28] based on the IPAQ guidelines [29]. Walking, moderate physical activity, and vigorous physical activity were scored at 2.3, 3.0 and 7.0 excess metabolic equivalents of tasks (METs), respectively [29]. Then, the time spent in each of the activities on a typical day was multiplied by the typical number of days doing the exercise and the respective MET scores to assess METs per week. A daily physical activity of less than 10 min was recorded as 0, and self-reported values of more to 1260 min per week (equivalent to an average of 3 h a day) were cut off at 1260 min according to the IPAQ guidelines [27]. Participants who answered “do not know” or “prefer not to answer” to any of the self-reported physical activity questions were excluded from the analysis. MET hours per week were categorized as low: <10.0; moderate: 10.0 to 49.9; and high: ≥50 MET hours/week) [29].

### 2.7. Statistical Analysis

The characteristics of the study population were described as the means with standard deviations (SDs) for continuous variables. Categorical variables were described as numbers and proportions. Comparisons between groups were performed using Student’s test for continuous variables. Pearson’s χ2 test was used for categorical variables. This study explored the association between social isolation and loneliness with estimated 10-year ASCVD risk levels and secondly with a high risk of CV (an estimated 10-year ASCVD risk of more than 7.5%). Associations between social isolation and loneliness with estimated 10-year ASCVD risk were examined with multiple linear regression models computing regression coefficients (*B*) and their 95% confidence intervals (95% CIs) and then with multiple logistic regression models with odds ratio (ORs) and 95% confidence intervals to estimate the 10-year ASCVD risk of more than 7.5%. First, the gender models were adjusted for age. Second, the gender models were adjusted for age, antidepressant medication, education, income level, couple, physical activity, Townsend deprivation quintiles, BMI, CKD, and triglycerides. These adjustments were justified by their relationship with ASCVD risk and CV risk: education [30], income [31], couple [32], physical activity [33], Townsend deprivation [34], BMI [35], CKD [36] and triglycerides [37].

The “no poor social isolation and no loneliness” participant group was considered as the reference group in the analyses. Statistics were calculated using SAS software (version 9.4; SAS Institute, Carry, NC, USA). A *p*-value < 0.05 was considered statistically significant.

## 3. Results

Among the 301,550 participants, social isolation affected 26,454 (8.77%) and loneliness affect 17,555 (5.82%).

Men presented a higher estimated 10-year ASCVD risk than women (8.63% vs. 2.65%, *p* < 0.001), a higher proportion of high education levels (38.44% vs. 36.23%, *p* < 0.001) and a higher proportion of high-income levels (31.37% vs. 25.29%, *p* < 0.001). Men also showed higher proportions of social isolation (9.13% vs. 8.45%, *p* < 0.001) and loneliness (6.11% vs. 5.56%, *p* < 0.001) (Table 1).

**Figure 2:**
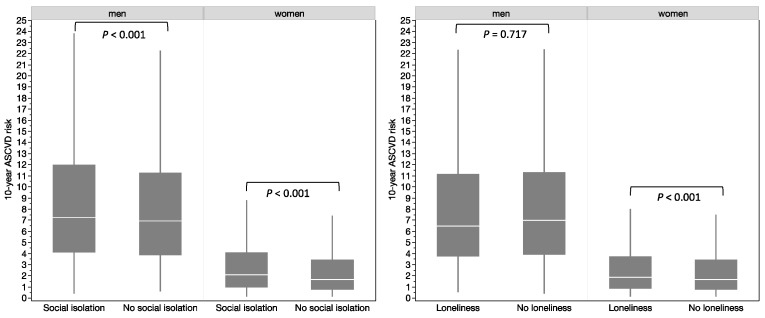
Estimated 10-year ASCVD risk according to gender and social isolation and loneliness status (white line: median; grey area: 25th to 75th percentile).

In both genders, participants with social isolation showed higher levels of ASCVD risk than the reference groups (for men with social isolation, the mean ASCVD risk = 9.26% vs. 8.57%, *p* < 0.001; for women with social isolation, the mean ASCVD risk was 3.23% vs. 2.60%, *p* < 0.001) (Figure 2). No univariable difference was observed for loneliness among men (mean ASCVD risk = 8.60% vs. 8.63%, *p* = 0.717), but a significant association among women was observed (mean ASCVD risk = 2.91% vs. 2.64%, *p* < 0.001) (Figure 2).

However, in both genders, participants with social isolation and loneliness showed lower high income and high educational level rates than the reference groups (*p* < 0.001).

When considering the overall population, both social isolation and loneliness showed significant associations with estimated 10-year ASCVD risk, as did social isolation (*B* = 0.14 (0.11; 0.17), *p* < 0.001) and not loneliness (*B* = 0.02 (−0.01; 0.05), *p* = 0.198), with a significant *p-*value for the interaction between social isolation and loneliness and gender (*p* < 0.001).

Compared with the reference group, men with social isolation were associated with a higher estimated 10-year ASCVD risk (*B* = 0.41 (0.37; 0.47), *p* < 0.001) in the age-adjusted model. Adjustment for all covariates did not affect this association (*B* = 0.21 (0.16; 0.26), *p* < 0.001). The same results were observed for analyses of high ASCVD risk with all covariates adjusted (OR = 1.14 [1.08–1.20], *p* < 0.001) (Table 2).

Compared with the reference group, men with loneliness were associated with a higher estimated 10-year ASCVD risk (*B* = 0.30 (0.24; 0.36), *p* < 0.001) in the age-adjusted model and after adjustment for all covariates (*B* = 0.08 (0.03; 0.14), *p* = 0.001). However, after adjustment for all covariates, no association between loneliness and high ASCVD risk was observed (*p* = 0.735). Loneliness and social isolation showed a significant interaction (*p* = 0.009) with ASCVD risk. When considering men with both social isolation and loneliness, the relationship with continuous ASCVD risk was higher than social isolation and loneliness alone (after adjustment for all covariates: *B* = 0.44 (0.28; 0.61), *p* < 0.001) but similar ORs with social isolation alone when considering an ASCVD risk of more than 7.5% (OR = 1.15 [1.02–1.29], *p* = 0.028) (Table 2).

In women, compared with the reference group, social isolation was associated with a higher estimated 10-year ASCVD risk in men (*B* = 0.22 (0.20; 0.24), *p* < 0.001) in the age-adjusted model. Adjustment for all covariates did not affect the association (*B* = 0.12 (0.10; 0.14), *p* < 0.001).

The same results were observed when I performed analyses of high ASCVD risk with all covariates adjusted (OR = 1.37 [1.27–1.49], *p* < 0.001) (Table 3).

Compared with the reference group, women with loneliness were associated with a higher estimated 10-year ASCVD risk (*B* = 0.08 (0.06; 0.11), *p* < 0.001) in the age-adjusted model but not after adjustment for all covariates (*p* = 0.217). This relationship remained insignificant after adjustment for all covariates with high ASCVD risk (*p* = 0.058). Loneliness and social isolation showed a significant interaction (*p* = 0.016) with ASCVD risk. When considering women with both social isolation and loneliness, the relationship with continuous ASCVD risk was higher than social isolation and loneliness alone (after adjustment for all covariates: *B* = 0.20 (0.12; 0.29), *p* < 0.001) and when considering an ASCVD risk of more than 7.5% (OR = 1.82 [1.52–2.16], *p* < 0.001) (Table 3).

## 4. Discussion

The main findings of this study were that social isolation was associated with a higher estimated 10-year ASCVD risk in both genders, but only loneliness was associated with a higher estimated 10-year ASCVD risk in men. A significant trend was found between loneliness and CV risk in women (*p* = 0.134).

In this study, I found that men showed a higher ASCVD risk than women, which is consistent with the literature [11,38,39,40,41]. As observed in previous studies, we found a significantly higher proportion of social isolation and loneliness in men than in women [42,43]. Moreover, my findings are consistent with the literature where social isolation and loneliness have been associated with CV diseases [3,7,11,13,44]. A previous study from the National Health and Nutrition Examination Survey among 2616 Americans showed that loneliness was associated with coronary heart disease (CHD) incidence in women but not in men [44]. The results of the English Longitudinal Study of Ageing (ELSA) showed that loneliness was associated with heart disease and stroke incidence [3,45]. Moreover, previous studies from the UK Biobank study showed that social isolation was associated with CHD and stroke [9], but another study found no association between loneliness and acute myocardial infarction and a significant association between social isolation, but not loneliness, with mortality [7].

According to previous studies, the relationship between social isolation and CV disease could be stronger than the association between loneliness and CV disease [7,11]. We observed similar findings in my study, where the combination of both social isolation and loneliness showed higher combined effects on CV risk than loneliness in men and both social isolation and loneliness in women.

Social isolation and loneliness are associated with diminutions in physical activity [46], higher proportions of tobacco smoking [47], poor diets [48], and the higher consumption of alcohol [49]. Having social isolation or loneliness may have indirect action in CV disease prevention by reducing good health behavior. Nevertheless, these interactions remain too complex to highlight and clearly explain.

Several social isolation and loneliness pathways that affect health have been identified [6]. It remains crucial to identify all these pathways to develop specific and personalized health policies [6]. Social isolation and loneliness are major risk factors for mental illnesses, such as depression [50], anxiety, and suicidal behavior [51]. Moreover, social isolation and loneliness are considered poor outcomes of CV diseases [6,51].

A possible biological pathway associating social isolation and loneliness with CV risk was found to be the immune system, which activates the inflammatory process [6,52]. Cytokine production was found to be associated with loneliness through the increased production of interleukin IL-6, IL-1bB, tumor necrosis factor alpha (TNF-alpha), fibrinogen, and monocyte chemoattractant protein 1 (MCP-1) [53]. Nevertheless, no association between CRP and loneliness has been observed [54]. In parallel to inflammation, oxidative stress could be another possible biological pathway to explain the relationship between loneliness and CV risk [55].

Loneliness interferes with cardiometabolic changes by increasing blood pressure [6,53], heart rate [53], vascular resistance [53] and hypertension [56]. The psychosocial stress generated by loneliness could change gut microbiota The activation of the hypothalamic pituitary adrenal (HPA) axis leads to decreases in microbial diversity [57], thus leading to an increased risk of CV disease [58].

Few studies have investigated the interaction between social isolation and loneliness. Nevertheless, loneliness activates the HPA axis and leads to increases in the perception of social isolation [50,59]. The HPA axis sets a “flight or fight” response into motion by producing cortisol, the stress hormone, at higher levels upon awakening [59]. Chronic stress leads to chronically increased cortisol rates, which are correlated with CV disease incidence [60].

### Strengths and Limitations

The major strength of the study was the large sample size of the UK Biobank cohort. The cross-sectional design limited the ability to establish a causality relationship, so reverse causation cannot be ruled out. The UK Biobank study presented a low 5.5% response rate, suggesting the possibility of participants’ bias. However, given the large sample size and high internal validity, these limitations were unlikely to affect the observed associations [61,62,63]. Moreover, the study investigation was focused on middle-aged UK participants, so my results cannot be generalized to other age and ethnic populations. Nevertheless, the UK Biobank uses standardized protocols to collect data. This standardization ensures the replication of data collection for all participants regardless of when, where and by whom it was performed and adds external validity to my findings. Nevertheless, my study had many limitations: socio-economic, medical history, comorbidity, and social isolation and loneliness data were collected with self-reported questionnaires or by physicians during medical examination in health centers. The adjustment of both income and education may be considered potential biases due their significant interaction relationships with ASCVD risk. No clear information about thyroid disease was collected in this study, so it cannot be considered a possible cofounding factor.

## 5. Conclusions

Both social isolation and loneliness and their combination can be considered potential added risk factors for CV risk. Thus, health policies should address these concepts in prevention campaigns, in addition to traditional risk factors. Further studies should be performed to investigate the gender differences in social isolation and loneliness and their implications in CV prevention and management. Moreover, specific information addressed to health professionals may be targeted [64] to promote the prevention and treatment of CV diseases among individuals with social isolation and loneliness.

## Figures and Tables

**Table 1:** Characteristics of the study population according to gender.

	Men *N* = 142,302		Women *N* = 159,248		*p*-Value
**Age (years) (mean, SD)**	56.08	8.16	55.49	7.97	<0.001
**Estimated 10-year ASCVD risk (%) (mean, SD)**	8.63	6.70	2.65	3.02	<0.001
**High level of ASCVD risk (>7.5%)**	65,626	46.12%	9624	6.04%	<0.001
**Physical activity**					<0.001
High	33,194	23.33%	30,749	19.31%	
Moderate	72,998	51.30%	85,003	53.38%	
Low	36,110	25.38%	43,496	27.31%	
**BMI (kg/m^2^) (mean, SD)**	27.65	4.11	26.82	5.05	<0.001
**BMI**					<0.001
High	33,783	23.74%	34,710	21.80%	
Moderate	71,379	50.16%	58,088	36.48%	
Low	37,140	26.10%	66,450	41.73%	
**Couple**	111,442	78.35%	109,783	68.96%	<0.001
**Education**					<0.001
High	54,698	38.44%	57,688	36.23%	
Moderate	61,500	43.22%	73,934	46.43%	
Low	26,104	18.34%	27,626	17.35%	
**Income**					<0.001
High	44,646	31.37%	40,272	25.29%	
Moderate	73,146	51.40%	83,464	52.41%	
Low	24,510	17.22%	35,512	22.30%	
**Townsend deprivation quintiles**					<0.001
Q1	29,276	20.57%	31,110	19.54%	
Q2	28,828	20.26%	31,527	19.80%	
Q3	28,307	19.89%	32,004	20.10%	
Q4	27,655	19.43%	32,623	20.49%	
Q5	28,236	19.84%	31,984	20.08%	
**Antidepressant medication**	6209	4.36%	13,967	8.77%	<0.001
**Antihypertensive medication**	28,179	19.80%	23,651	14.85%	<0.001
**Diabetes**	9835	6.91%	7115	4.47%	<0.001
**Black people**	461	0.32%	750	0.47%	<0.001
**Current smoking**	16,685	11.74%	13,832	8.70%	<0.001
**HDL cholesterol (mmol/L) (mean, SD)**	1.29	0.31	1.61	0.37	<0.001
**Total cholesterol (mmol/L) (mean, SD)**	5.59	1.09	5.88	1.11	<0.001
**Triglycerides (mmol/L) (mean, SD)**	1.97	1.15	1.52	0.84	<0.001
**SBP (mmHg) (mean, SD)**	138.97	15.84	126.76	17.41	<0.001
**eGFR (mL/min/1.73 m^2^) (mean, SD)**	98.15	26.65	132.67	33.65	<0.001
**Chronic kidney disease**	5723	4.02%	913	0.57%	<0.001
**Social isolation**	12,999	9.13%	13,455	8.45%	<0.001
**Loneliness**	8698	6.11%	8857	5.56%	<0.001
**Both social isolation and loneliness**	2213	1.56%	1722	1.08%	<0.001

SD: standard deviation.

**Table 2:** Multiple linear and logistic regression models for estimated 10-year ASCVD risk among men (all covariates adjusted: age, education, income level, physical activity, couple, antidepressant medication, Townsend deprivation index, BMI, triglycerides, and CKD). The reference was the reference group.

	Continuous Estimated 10-Year ASCVD Risk				Estimated 10-Year ASCVD Risk Superior to 7.5%			
Men	Age-Adjusted Model		All Covariate-Adjusted Model		Age-Adjusted Model		All Covariate-Adjusted Model	
	Beta (95% CI)	*p*-Value	Beta (95% CI)	*p*-Value	OR 95% CI	*p*-Value	OR 95% CI	*p*-Value
Loneliness	0.30 (0.24; 0.36)	<0.001	0.08 (0.03; 0.14)	0.001	1.19 [1.12–1.26]	<0.001	1.01 [0.95–1.07]	0.735
Social isolation	0.41 (0.37; 0.47)	<0.001	0.21 (0.16; 0.26)	<0.001	1.30 [1.24–1.36]	<0.001	1.14 [1.08–1.20]	<0.001
Both social isolation and loneliness *	0.90 (0.73; 1.08)	<0.001	0.44 (0.28; 0.61)	<0.001	1.54 [1.38–1.72]	<0.001	1.15 [1.02–1.29]	0.028

* Analysis was performed independently of loneliness and social isolation.

**Table 3:** Multiple linear and logistic regression models for estimated 10-year ASCVD risk among women (all covariates adjusted: age, education, income level, physical activity, couple, antidepressant medication, Townsend deprivation index, BMI, triglycerides, and CKD). The reference was the reference group.

	Continuous Estimated 10-Year ASCVD Risk				Estimated 10-Year ASCVD Risk Superior to 7.5%			
Women	Age-Adjusted Model		All Covariate-Adjusted Model		Age-Adjusted Model		All Covariate-Adjusted Model	
	Beta (95%CI)	*p*-Value	Beta (95%CI)	*p*-Value	OR 95% CI	*p*-Value	OR 95% CI	*p*-Value
Loneliness	0.08 (0.06; 0.11)	<0.001	0.01 (−0.01; 0.03)	0.217	1.25 [1.14–1.37]	<0.001	1.09 [0.99–1.20]	0.058
Social isolation	0.22 (0.20; 0.24)	<0.001	0.12 (0.10; 0.14)	<0.001	1.62 [1.51–1.73]	<0.001	1.37 [1.27–1.49]	<0.001
Both social isolation and loneliness *	0.39 (0.30; 0.48)	<0.001	0.20 (0.12; 0.29)	<0.001	2.39 [2.02–2.83]	<0.001	1.81 [1.52–2.16]	<0.001

* Analysis was performed independently of loneliness and social isolation.

## Data Availability

The data that support the findings of this study are available on request from the corresponding author. The data are not publicly available due to privacy or ethical restrictions.
